# Anxiety Disorders are Associated with Reduced Heart Rate Variability: A Meta-Analysis

**DOI:** 10.3389/fpsyt.2014.00080

**Published:** 2014-07-11

**Authors:** John A. Chalmers, Daniel S. Quintana, Maree J.-Anne Abbott, Andrew H. Kemp

**Affiliations:** ^1^School of Psychology, University of Sydney, Sydney, NSW, Australia; ^2^Brain and Mind Research Institute, University of Sydney, Sydney, NSW, Australia; ^3^NORMENT, KG Jebsen Centre for Psychosis Research, Institute of Clinical Medicine, University of Oslo, Oslo, Norway; ^4^Division of Mental Health and Addiction, Oslo University Hospital, Oslo, Norway; ^5^University Hospital and Faculty of Medicine, University of São Paulo, São Paulo, Brazil; ^6^Discipline of Psychiatry, University of Sydney, Sydney, NSW, Australia

**Keywords:** heart rate variability, anxiety, anxiety disorders, meta-analysis, treatment, cardiovascular disease

## Abstract

**Background:** Anxiety disorders increase risk of future cardiovascular disease (CVD) and mortality, even after controlling for confounds including smoking, lifestyle, and socioeconomic status, and irrespective of a history of medical disorders. While impaired vagal function, indicated by reductions in heart rate variability (HRV), may be one mechanism linking anxiety disorders to CVD, prior studies have reported inconsistent findings highlighting the need for meta-analysis.

**Method:** Studies comparing resting-state HRV recordings in patients with an anxiety disorder as a primary diagnosis and healthy controls were considered for meta-analysis.

**Results:** Meta-analyses were based on 36 articles, including 2086 patients with an anxiety disorder and 2294 controls. Overall, anxiety disorders were characterized by lower HRV [high frequency (HF): Hedges’ *g* = −0.29. 95% CI: −0.41 to −0.17, *p* < 0.001; time domain: Hedges’ *g* = −0.45, 95% CI: −0.57 to −0.33, *p* < 0.001] than controls. Panic disorder (*n* = 447), post-traumatic stress disorder (*n* = 192), generalized anxiety disorder (*n* = 68), and social anxiety disorder (*n* = 90), but not obsessive–compulsive disorder (*n* = 40), displayed reductions in HF HRV relative to controls (all *p*s < 0.001).

**Conclusion:** Anxiety disorders are associated with reduced HRV, findings associated with a small-to-moderate effect size. Findings have important implications for future physical health and well-being of patients, highlighting a need for comprehensive cardiovascular risk reduction.

## Introduction

Anxiety disorders are the most prevalent psychiatric disorders (1), and one of the most costly (2). Anxiety disorders also increase risk of cardiovascular disease (CVD) three- to fourfold, after accounting for sex, substance use, and depression (3, 4) while risk of cardiac mortality is increased twofold (5–7). Here, we examine the impact of the anxiety disorders on a psychophysiological marker of health and well-being, heart rate variability (HRV). Previous studies are characterized by contradictory reports, highlighting the need for a meta-analysis of previously published findings.

Heart rate variability is the fluctuation of heart period over time, commonly measured by electrocardiogram (ECG), and is an important marker of psychological well-being, general cardiovascular health, and is a major predictor of mortality (8–10). The mechanism underpinning the relationship between mental and physical health may, in part, relate to impairment in vagal nerve activity leading to a dysregulation of inflammatory processes (11, 12). Reductions in resting-state HRV reflect decreases in vagal output. We have now published several meta-analyses reporting HRV reductions in depression (13) and alcohol dependence (14). To date, no meta-analysis has been published on HRV and the anxiety disorders; this is a surprising observation considering that anxiety is an early marker of cardiovascular risk and a robust predictor of CVD and sudden cardiac death, independent of demographic risk factors, biological risk factors, and health behaviors (5, 6, 15).

There are a variety of reasons to expect that HRV will be reduced in the anxiety disorders. According to the neurovisceral integration model (16), efferent nerve fibers from the pre-frontal cortex moderate parasympathetic activity and vagal nerve inhibition of cardiac activity, where proper vagus nerve regulation mediates inflammatory processes that lead to a variety of pathologies, including type-II diabetes (17), clinical depression (18), coronary heart disease (19), and neurodegenerative diseases (20). The neurovisceral integration model also characterizes a detailed network of specific neural structures that enable humans to adaptively respond to environmental, physiological, behavioral, cognitive, and emotional influences. In this model, a healthy autonomic nervous system is characterized by high levels of adaptive variability, or rheostasis (21, 22). An illustration of this point can be seen in the observation that a healthy cardiorespiratory system is characterized by complex oscillation patterns in heart period (or high HRV) whereas a diseased system displays little to no variability (9). A key feature of the neurovisceral integration model is the central autonomic network (CAN), a network of brain regions that coordinates autonomic, endocrine, and behavioral responses in goal directed action and in adaptation to environmental challenges. The integrity of this network is compromised in anxiety; sympathoexcitatory responses are unable to be effectively inhibited, leading to behavioral inflexibility. The neurovisceral integration model also links hypervigilance and worry – features observed in all the anxiety disorders (23) – to reductions in HRV. Chronic reductions in resting-state HRV appear to be associated with worry (24–27) and pathological worry has been implicated in the development of CVD and CVD risk factors, where HRV is one mechanism of cardiopathogenesis (28). Further, HRV reductions may also be observed during phasic forms of anxiety, such as panic symptomatology (29–31). Given that many treatments aim to address these features of anxiety, it may be that successful treatments may positively impact on autonomic functioning. Interventions that improve HRV (e.g., exercise) may also help to ameliorate hypervigilance and worry (32–34). These bidirectional associations are supported by reciprocal connections between brain and body (16, 35).

Many studies have reported reduced HRV in people with anxiety disorders. The majority of studies in the literature have focused on panic disorder (PD); over 20 studies have been conducted to date on HRV in PD patients. Researchers have also reported on the impact of post-traumatic stress disorder (PTSD), generalized anxiety disorder (GAD), obsessive–compulsive disorder (OCD), social anxiety disorder (SAD), specific phobias, and mixed/grouped anxiety disorders on HRV, with equivocal results (see Table 1 below for a summary). The reason for the inconsistency in results across studies is unclear, although two possible explanations present themselves: small sample size and common confounds including psychiatric and medical co-morbidity, as well as medication use.

**Table 1:** **Summary of studies reporting comparisons in HRV between patients with anxiety disorders and controls**.

Study	HRV measures	Participants with anxiety disorder	Healthy control subjects	Sig. diff between groups	Major finding
**PANIC DISORDER (PD)**					
Alvargenga et al. (36)^a^	HF, LF	25	20	+	Lower HF power in PD compared with controls
Chang et al. (37)^a^	HF, LF, TD	48	202	+	Lower HRV in PD patients compared with controls in all measures
Cohen et al. (30)^a^	HF, LF	11	25	+	Lower HF power and higher LF power in PD relative to controls
Garakani et al. (31)^a^	HF, LF, TD	43	11	+	Lower PNN 50% in PD relative to controls. HF and LF power non-significant between groups
Ito et al. (38)^a^	HF, LF	8	13	−	No differences in HF and LF power between PD and controls
Kang et al. (39)^a^	HF, LF, TD	45	30	+	Lower HF power and higher LF power in PD relative to controls. All TD measures non-significant
Kikuchi et al. (40)^a^	HF, LF, TD	17	15	−	No differences between PD and controls on all measures
Lavoie et al. (41)^a^	HF, LF	20	22	−	LF and HF power non-significant (but lower LF/HF ratio in PD relative to controls)
Martinez et al. (42)^a^	HF, LF, TD	30	10	+	Higher LF power and LF/HF ratio in PD relative to controls; lower PNN50 in PD
McCraty et al. (43)^a^	HF, LF	38	38	+	LF/HF ratio and LF power lower in PD relative to controls; higher HF power in PD
Melzig et al. (44)^a^	TD	9	15	+	Lower RMSSD in PD relative to controls
Middleton and Ashby (45)^a^	TD	12	12	−	No differences in HF power between groups. Reduced HR standard deviation in PD
Pittig et al. (29)^a^	HF	39	39	+	Lower HF HRV in PD patients
Prasko et al. (46)^a^	HF, LF	52	104	+	Lower LF/HF ratio and SDNN in PD relative to controls
Slaap et al. (47)^a^	HF	24	24	−	No differences on any HRV measure between PD and controls
Wang et al. (48)^a^	HF, LF	27	20	−	No difference between PD patients and controls
Wise et al. (49)^a^	TD	30	20	+	Lower R–R variance in PD compared to controls
Yeragani et al. (50)^a^	TD	21	21	+	Lower HR standard deviation in PD relative to controls
Yergani et al. (51)^a^	HF, LF	6	11	−	No differences between PD and controls on all measures at baseline
Asmundson and Stein (52)^b^	RSA	15	15	−	No differences in parasympathetic nervous system function between PD and controls
Blelchert et al. (53)^b^	RSA	26	32	−	No differences in parasympathetic nervous system function between PD and controls
Klein et al. (54)^b^	HF	10	14	+	Lower HF power in PD relative to controls
Petrowski et al. (55)^b^	TD	14	14	−	No differences in TD measures between PD and controls
Yergani et al. (56)^b^	TD	29	23	−	Lower absolute ULF in PD relative to controls
**POST-TRAUMATIC STRESS DISORDER (PTSD)**					
Agorastos et al. (57)^a^	TD	7	8	−	Reduced HRV in PTSD patients, but did not reach significance
Cohen et al. (58)^a^	HF, LF	9	9	+	Lower HF and higher LF power in PTSD relative to controls
Hauschildt et al. (59)^a^	HF, LF, TD	26	18	+	Lower HF power and RMSSD in PTSD relative to controls. LF power non-significant
Keary et al. (60)^a^	HF, LF	20	20	−	No differences at baseline between PTSD and controls
Lakusic et al. (61)^a^	HF, LF, TD	34	34	+	Lower HF power and RMSSD in PTSD relative to controls; higher LF power in PTSD
Norte et al. (62)^a^	TD	19	16	+	Lower HRV in PTSD patients
Shah et al. (63)^a^	HF, LF	31	385	+	Lower HF and LF HRV in PTSD patients
Tucker et al. (64)^a^	HF, LF	13	32	+	Higher LF power and LF/HF ratio in PTSD relative to controls
Wahbeh and Oken (65)^a^	HF, LF	52	29	+	Reduced HF and LF in PTSD patients relative to controls
Blechert et al. (53)^b^	RSA	23	32	+	Lower parasympathetic nervous system function in PTSD relative to controls
Cohen et al. (30)^b^	HF, LF, TD	14	25	+	Lower HF and higher LF power in PTSD relative to controls
Cohen et al. (66)^b^	HF, LF	16	16	+	Lower HF and higher LF power in PTSD relative to controls
Shaikh Al Arab et al. (67)^b^	TD	7	11	+	Lower RMSSD in PTSD relative to controls
**GENERALIZED ANXIETY DISORDER (GAD)**					
Hammel et al. (68)^a^	HF, LF, TD	16	19	−	No differences in any HRV measure between GAD patients and controls
Lyonfields et al. (24)^a^	TD	15	15	+	Lower mean successive differences in GAD relative to controls
Pittig et al. (29)^a^	HF	26	39	−	Borderline lower HF HRV in GAD patients (*p* = 0.06)
Thayer et al. (25)^a^	HF, LF, TD	34	32	+	Lower HF power in GAD compared to control
Kollai and Kollai (69)^b^	RSA	19	18	+	Lower RSA in GAD compared to control
**OBSESSIVE–COMPULSIVE DISORDER (OCD)**					
Pittig et al. (29)^a^	HF	17	39	+	Lower HF HRV in OCD patients
Slaap et al. (47)^a^	HF, LF	26	24	−	No differences between OCD and controls
**SPECIFIC PHOBIA**					
Bornas et al. (70)^a^	HF, LF, TD	61	58	+	Difference in TD HRV between flight phobics and controls, but not HF and LF HRV
**SOCIAL ANXIETY DISORDER**					
Alvares et al. (71)^a^	HF, LF, TD	53	53	+	Significant reductions in HF HRV and RMSSD for social phobics relative to controls
Gaebler et al. (72)^a^	HF	21	21	+	Lower HF HRV in social phobics
Pittig et al. (29)^a^	HF	29	39	+	Lower HF HRV in social phobics
Asmundson and Stein (52)^b^	RSA	15	15	−	No differences in parasympathetic activity between social phobics and controls
**MIXED ANXIETY DISORDER**					
Einvik et al. (73)^a^	HF, LF, TD	20	231	−	No differences in HF or LF power, or SDNN between anxiety patients and controls
Licht et al. (74)^a^	TD, RSA	1159	616	+	Lower SDNN and RSA in anxiety patients relative to controls. Effect disappears when controlling for psychotropic use
Martens et al. (75)^a^	HF, LF, TD	7	59	+	Lower RMSSD in anxiety patients relative to controls

*TD, time domain; HF, high frequency; LF, low frequency; RSA, respiratory sinus arrhythmia*.

*^a^Study included in meta-analysis*.

*^b^Study excluded from meta-analysis*.

Overall, the impact of anxiety and their treatments on HRV is mixed. While many studies report significant HRV reductions in anxiety disorder patients, some report no significant findings. This pattern of results could be considered to reflect an underlying negative “true” relationship, a hypothesis we set to test meta-analytically. A narrative review published 7 years ago (21) comprehensively reviewed studies on the impact of the anxiety disorders on HRV, concluding that “reports of aberrant HRV features of panic outnumber negative findings by a ratio of greater than 6:1” [(21) p.191]. A limitation of this approach is that it does not take into account the magnitude of the effects, whether effect sizes are consistent across studies, or the possibility that non-significant outcomes may have been afflicted by insufficient power. Conversely, meta-analysis provides a robust statistical method of synthesizing effect sizes across studies, and is a valuable tool for clarifying contradictory findings. In summary, there is a need for an up-to-date systematic review of the literature given the important implications that reductions in HRV may have over the long term.

In the present study, we sought to determine the impact of anxiety on resting-state HRV, a psychophysiological marker of health and well-being, minimizing variation across studies. To our knowledge, the present report is the first to address this question using meta-analytic methodology. Specifically, we examine whether patients with any anxiety disorder exhibit reductions in HRV relative to healthy controls, and determine the size of this effect across specific anxiety disorders. We hypothesized that anxiety patients overall would display reductions in HRV relative to healthy participants.

## Materials and Methods

### Search criteria

The search strategy followed guidelines outlined in the preferred reporting items for systematic reviews and meta-analyses (PRISMA) (76) statement. Peer-reviewed studies were located in Embase, MEDLINE, and PsychINFO, with all relevant combinations of the following words and phrases: “heart rate variability,” “HRV,” “vagal,” “parasympathetic,” “autonomic nervous system,” “generalized anxiety disorder,” “generalized anxiety disorder,” “GAD,” “obsessive-compulsive disorder,” “obsessi*,” “compulsi*,” “OCD,” “panic disorder” “panic disorder with* agoraphobia,” “PD,” “post-traumatic stress disorder,” “PTSD,” “acute stress disorder,” “social anxiety disorder,” “SAD,” “social phobi*,” “specific phobia,” and “anxi*.” In addition to these electronic searches, each report’s citation list was examined for additional studies. The search was performed during October 2013, with no limitation on time-period. The inclusion criteria were: (1) the comparison of HRV in patients with a diagnosis of an anxiety disorder as defined by DSM-III (77), DSM-III-R (78), DSM-IV (79), DSM-IV-TR (80), or ICD-10 (81), and a control group free from psychiatric diagnosis; (2) satisfactory reporting of statistics (i.e., mean, SD, *p, t, r*, or *F* value); and (3) the study was written in English. No limitations were made concerning medication status, physical illness including CVD, or disorder co-morbidity; however these factors were included in follow-up moderator analyses. All potentially relevant manuscripts were independently reviewed by two investigators (JAC and DSQ) and areas of disagreement or uncertainty were adjudicated by a third investigator (AHK).

### Procedure

Meta-analyses were conducted to answer the primary research question of whether patients with anxiety disorders exhibit reductions in resting-state HRV relative to controls, and to determine the size of this effect across specific anxiety disorders. HRV is recorded under short-term (2 min to 1 h) or long term (24 h) conditions, both of which provide reliable indicators of autonomic functioning and are robust predictors of mortality (8) and future cardiac events (82). A variety of HRV measures are reported in the literature, which generally fall under time domain (TD) and frequency domain (83–85). A third domain of HRV is respiratory sinus arrhythmia (RSA), which reflects parasympathetic nervous system activity by indexing the coupling of heart period and respiration. The TD measures extracted for analysis included RMSSD, SDNN, pNN50, and SDANN. If more than one TD measure was reported RMSSD was given preference, followed by SDNN, pNN50 then SDANN. RMSSD was given preference as it closely represents parasympathetic activity and is highly correlated with the high frequency (HF) HRV component. Of the frequency domain HRV measures, HF HRV reflects parasympathetic (vagal) nervous system output (86) and is the most commonly reported measure of HRV in the anxiety literature. The frequency domain measures extracted were high and low frequency (LF) HRV. There is an ongoing debate as to the interpretation of LF HRV (85, 87), and consequently there are sound arguments against the utility and meaningfulness of the oft reported measure LF/HF ratio (88, 89). As such, this measure was not extracted. There are also a number of non-linear methods used to evaluate HRV (85); however, anxiety studies using non-linear methods [e.g., Ref. (71, 90)] have not accumulated to the point that meta-analysis is feasible. Using moderator analyses, we examined possible impacts of known confounds, including medical and psychiatric co-morbidity as well as medication status. Moderator analyses also examined the potential impact of study characteristics (i.e., diagnostic criteria) on HRV parameters. Finally, while short and long-term assessments of HRV have been found to be significantly related to one another, the reported correlation coefficients are rather weak (91). Thus, moderator analyses also compared studies reporting short-term versus long-term recordings to examine if recording method impacted the results.

### Meta-analysis statistics

Meta-analyses were based on a single effect size of a standardized mean. Values were transformed from available statistics (e.g., means and SDs) to determine a standardized effect size, Hedges’ *g*, using the computer software package comprehensive meta-analysis (92). Hedges’ *g* is related to Cohen’s *d* and can be interpreted using the same conventions: small (0.2), medium (0.5), and large (0.8) (93). An added benefit of Hedges’ *g* is correction for biases found in small sample sizes. The random-effects model was applied in the present meta-analysis, thereby adopting a conservative approach that assumes true effect size may vary from study to study, allowing results to be generalized to populations beyond the study samples (94). To measure homogeneity of effect sizes across studies, the *Q* statistic was examined. A significant *Q* statistic is indicative of dissimilar effect sizes across studies; suggesting that methodological or population sample differences might be introducing variance in findings across studies (95). To complement the *Q* test, we also calculated the *I*^2^ statistic, which provides an index of the degree of heterogeneity across studies, where *I*^2^ signifies the percentage of the total variability in effect sizes due to between-studies variability, and not due to sampling error within studies. Percentages of around 25% (*I*^2^ = 25), 50% (*I*^2^ = 50), and 75% (*I*^2^ = 75) may be interpreted as low, medium, and high heterogeneity, respectively (96). Begg’s adjusted rank correlation test (97) and Egger’s regression test (98) were used to assess publication bias. The Duval and Tweedie “Trim and Fill” procedure (99) was used to adjust for any suspected publication bias using a random-effects model. This procedure imputes the “missing” studies and recalculates the overall effect size with the inclusion of hypothetical studies.

## Results

### Included studies

**Figure 1:**
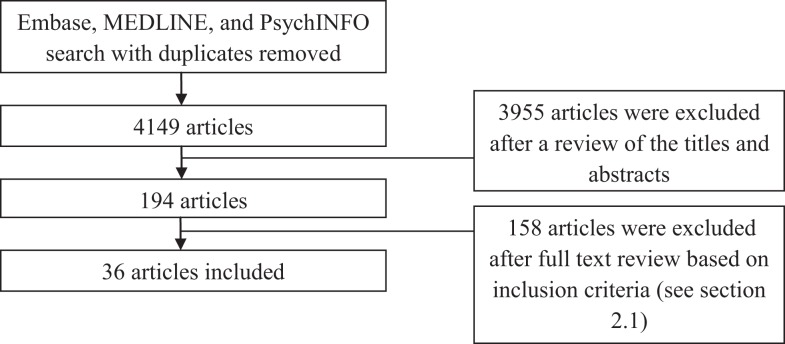
**Selection of articles for inclusion**. Note: diagnostic grouping of studies do not add to 36 due to some studies reporting on multiple diagnoses.

The search of electronic databases for HRV research in anxiety revealed 4149 studies as at October 2013; these were reduced to 194 after examining study abstracts and then to 36 after reviewing the study methods (Figure 1).

### Impact of anxiety disorders on HRV

High frequency HRV was reduced in participants with any anxiety disorder, regardless of specific diagnosis, relative to healthy controls [Hedges’ *g* = −0.29 (−0.41 to −0.17); SE = 0.06; *p* < 0.001], a finding associated with a small effect size. Egger’s regression test (*p* = 0.43) indicated no evidence of publication bias. The *Q* statistic was significant (*Q* = 51.39, *p* = 0.002) indicating study heterogeneity (*I*^2^ statistic = 35.8%). To examine findings in further detail, we examined effect sizes in specific anxiety disorders (see Table 2, which displays the summary effect sizes for each disorder; and Supplementary Material, which displays the relevant forest plots for each analysis.). HF HRV was significantly reduced in patients with GAD and SAD, findings associated with a moderate effect size, as well as in those with PD and PTSD, findings associated with a small effect size. Findings for OCD, specific phobia, and those studies in which anxiety disorders were grouped were all non-significant.

**Table 2:** **Meta-analysis results of HRV in specific anxiety disorders**.

Meta-analysis performed	No. of data sets	Number of anxious participants	Number of control participants	Comparison of anxious and control participants		
				Effect size (95% CI)	SE of summary effect size	*p* value
All disorders
Time domain HRV^a^	20	1615	1402	−0.70 (−1.45 to −0.05)	0.38	0.07
HF HRV	34	915	1659	−0.29 (−0.41 to −0.17)	0.06	<0.001
LF HRV	22	715	1115	−0.08 (−0.31 to 0.15)	0.09	0.49
Panic disorder
Time domain HRV	8	264	243	−0.41 (−0.68 to −0.15)	0.13	0.002
HF HRV	16	437	520	−0.22 (−0.42 to −0.02)	0.10	0.030
LF HRV	12	360	426	−0.11 (−0.47 to 0.25)	0.18	0.544
Post-traumatic stress disorder
Time domain HRV	4	86	76	−0.69 (−1.00 to −0.38)	0.16	<0.001
HF HRV	7	192	525	−0.29 (−0.58 to −0.001)	0.15	0.049
LF HRV	6	183	516	−0.04 (−0.51 to 0.42)	0.24	0.854
Generalized anxiety disorder
Time domain HRV	3	65	66	−0.55 (−0.89 to −0.21)	0.18	0.002
HF HRV	3	68	90	−0.56 (−0.87 to −0.25)	0.16	<0.001
LF	1	16	19	0.50 (−0.16 to 1.16)	0.34	0.140
Obsessive–compulsive disorder
HF HRV	2	40	63	−0.28 (−0.84 to 0.28)	0.29	0.328
LF HRV	1	26	24	−0.08 (−0.63 to 0.47)	0.28	0.773
Social anxiety disorder
Time domain HRV	1	53	53	−0.40 (−0.79 to −0.02)	0.20	0.038
HF HRV	3	90	113	−0.47 (−0.74 to −0.20)	0.14	0.001
LF HRV	1	53	53	−0.25 (−0.63 to 0.13)	0.19	0.205
Specific phobia
Time domain HRV	1	61	58	−0.38 (−0.74 to −0.02)	0.18	0.037
HF HRV	1	61	58	−0.05 (−0.41 to 0.31)	0.18	0.784
LF HRV	1	61	58	−0.05 (−0.41 to 0.31)	0.18	0.782
Mixed anxiety
Time domain HRV^b^	3	1086	906	−1.52 (−4.13 to 1.08)	1.33	0.251
HF HRV	2	27	290	−0.24 (−0.85 to 0.37)	0.31	0.442

*^a^Licht et al. (74) included, however, this study was not included in the final analysis. Without the inclusion of this large outlier, overall TD effect becomes significant [Hedges’ *g* = −0.45 (−0.57 to −0.33); SE = 0.06; *p* < 0.001]*.

*^b^Licht et al. (74) included*.

Moderator analysis revealed no significant differences across anxiety disorder diagnoses (*Q* = 6.83; *p* = 0.34). There was no evidence of heterogeneity between studies using different diagnostic criteria including DSM-III, DSM-IV, or ICD-10 (*Q* = 0.85, *p* = 0.65), nor was there heterogeneity for use of medication (*Q* = 0.1, *p* = 0.75), co-morbid psychiatric diagnoses (*Q* = 0.29, *p* = 0.59), or co-morbid medical disorders (*Q* = 0.004, *p* = 0.95). No evidence of heterogeneity between studies using short-term versus long-term recordings was obtained either (*Q* = 2.92, *p* = 0.09).

Low frequency HRV did not differ between participants with any anxiety disorder and controls [Hedges’ *g* = −0.08 (−0.31–0.15); SE = 0.09; *p* = 0.49]. Egger’s regression test (*p* = 0.001) indicated evidence of publication bias and the *Q* statistic was significant (*Q* = 85.02, *p* < 0.001) (*I*^2^ statistic = 75.3%). However, LF HRV did not differ for any anxiety disorder relative to healthy controls (Table 2). Moderator analyses revealed that LF HRV did not differ across anxiety disorders (*Q* = 3.73, *p* = 0.59); nor was there any evidence of heterogeneity from other factors.

Time domain HRV was reduced in participants with any anxiety disorder relative to controls [Hedges’ *g* = −0.7 (−1.45– 0.05); SE = 0.38; *p* = 0.07], a finding associated with a moderate effect size. While this finding was at trend levels, the overall *Q* statistic was significant (*Q* = 1027.39, *p* < 0.001) and the *I*^2^ statistic was 98.2% indicating high variance between studies, highlighting a need to further inspect these data. Egger’s regression test (*p* < 0.001) also indicated evidence of publication bias. After the exclusion of a clear outlier (74) (see Supplementary Material), HRV was significantly reduced in those with anxiety disorders relative to healthy controls [Hedges’ *g* = −0.45 (−0.57 to −0.33); SE = 0.06; *p* < 0.001], and there was no longer evidence of heterogeneity between studies (*Q* = 18.46, *p* = 0.43, *I*^2^ statistic = 2.5%). Egger’s regression test still revealed evidence of publication bias (*p* = 0.01). TD HRV was significantly reduced in patients with PTSD and GAD, findings associated with a moderate effect size, as well as in PD, SAD, and Specific Phobias, findings associated with a small effect size (see Table 2). Follow-up moderator analyses revealed no difference in summary effect sizes between disorders (*Q* = 2.8, *p* = 0.73). No evidence of heterogeneity from other factors was observed.

## Discussion

Meta-analysis revealed that anxiety disorders are associated with significant reductions in HRV, effects associated with a small-to-moderate effect size. Importantly, medication use and medical and psychiatric co-morbidity did not impact these findings. The present study revealed that PD, PTSD, GAD, and SAD displayed significant reductions in TD and HF HRV, findings associated with a moderate effect size. Specific phobias also displayed reductions in TD HRV, although these findings were associated with a small effect size. Effects relating to OCD and studies that grouped anxiety disorders were not associated with significant reductions. Further, our results suggest that anxiety disorders do not adversely impact on LF, perhaps highlighting the specificity of effects on the PNS, which is better reflected in HRV measures including RMSSD and HF. Considering that reductions in HRV predicts adverse future outcomes including CVD and sudden cardiac death (9, 10, 15), our findings have important implications for future physical health of patients with a diagnosis of anxiety, and PD, PTSD, GAD, and SAD in particular.

There are a number of possible explanations why OCD, in contrast with the other anxiety disorders, was not associated with reductions in HRV. First, the sample size was limited (*n* = 40). Second, there was inconsistency in reports between the two studies reporting on HRV and OCD. Slaap et al. (47) found that HF HRV parameters did not differ between patients and controls. Pittig et al. (29), however, was the first study to report reduced HF HRV in OCD patients relative to controls. As the authors noted, it is possible that results in the latter study could be accounted for by psychotropic medication use (11 of 17 OCD patients reported current medication use). The inconsistent results cited above mirror the inconsistencies in results on OCD patients and other indices of autonomic activity (100–103). In sum, more research is needed before firm conclusions can be drawn on cardiac autonomic functioning in these patients.

Anxiety, in all its forms, can be seen as a failure of inhibition involving reduced capacity to inhibit cognitive (e.g., apprehension, vigilance, and worry), affective (e.g., panic), behavioral (e.g., avoidance), and physiological (e.g., increased HR) responses, leading to reduced vagal outflow and lowered HRV. The prominent reductions in HRV observed here (with the exception of OCD) are in line with previous theoretical models. The neurovisceral integration model (16, 21) highlights a role for the prefrontal cortex in inhibitory function via a vagally mediated pathway, which can be indexed by HRV. In addition, the polyvagal theory (35) highlights a role for the myelinated vagus in promoting social engagement and communication, and cultivating relaxed behavioral states by inhibiting sympathetic tone on the heart and dampening the hypo-pituitary–adrenal axis. When the environment is perceived as safe, vagal outflow increases, promoting regeneration, homeostatic functions, and social behavior. Critically, impairment of these neural processes leads to a difficulty in detecting whether environments are safe or whether people are trustworthy, which in turn may play a role in the development of the anxiety disorders. Polyvagal theory may therefore help explain the link between anxiety and reduced capacity for inhibition as well as reduced social engagement characteristic of individuals with anxiety.

Our current findings have important implications for the established link between reduced HRV, anxiety, and health. All anxiety disorders exhibit greater threat-related attentional biases relative to controls (23). This perseverance results in chronically high levels of corticotropin releasing factor (a hormone and neurotransmitter released in response to stress) and high basal levels of cortisol, leading to chronically withdrawn parasympathetic activity (i.e., low vagal tone) (104). Additionally, chronic worriers may also display poor cardiac autonomic regulation in response to non-threatening cues (105). It is also possible that impaired vagal function, which usually plays an important role in regulating the hypothalamic–pituitary–adrenal axis (106), leads to heightened activation of stress responses. The inability to disengage from threat detection heightens activation of the sympathetic nervous system underpinned by a chronic withdrawal of parasympathetic activity (and long-term reductions in HRV). This chronicity may in turn contribute to chronic withdrawal of the parasympathetic nervous system, and subsequent impairment in the cholinergic anti-inflammatory reflex (107), leading to an increase in a host of conditions including diabetes, obesity, and CVD (9, 10). A prospective study on worry and future cardiac health has reported that high levels of worry alone increase the risk of future myocardial infarction two to threefold (108). Given the clear impact of chronic worry on future cardiac health, it is especially concerning given that anxiety patients may suffer for as long as 5–10 years before diagnosis and treatment (109). Given the potential health implications of suffering from an anxiety disorder, there are clear implications concerning the importance of discovering whether successful treatments have been shown to increase HRV in anxiety disordered patients.

Previous research concerning the question of the impact of treatment for anxiety on HRV can broadly be grouped into studies that investigated the impact of psychotropic and non-psychotropic therapies. Concerning psychotropic treatments, two studies have investigated the impact of pharmacological intervention in isolation on HRV in anxiety patients. First, Tucker et al. (110) examined the effect of paroxetine (an SSRI) administration on HRV in 17 PD patients. After 4 weeks of 20 mg of paroxetine daily, parasympathetic activity was significantly increased in patients. This finding stands in contrast to reports that SSRI treatments for major depression, including paroxetine, have no impact on HRV (13). Further, the study reports only normalized units of HF HRV, a measure difficult to interpret when not reported alongside raw HF values (88). Thus, the effect of SSRIs in isolation on HRV in anxiety patients has yet to be properly studied, however, we can predict the effect will be similar to the effect of SSRIs in treating major depression, that is, negligible (13). Second, Baker et al. (111) examined the effect of clonazepam (a benzodiazepine) administration on HRV relative to placebo in 27 PD patients. Ten patients received clonazepam while 17 received a placebo. Compared with placebo, patients receiving clonazepam showed a significant decrease in HRV for all time (SDANN, SDNN) and frequency (LF, HF) measures from baseline to 4 weeks (all *p*s < 0.05). Treatment response was not correlated with HRV. This result is consistent with previous studies observing reductions in HF HRV following benzodiazepine administration (112–114).

The non-psychotropic treatments for anxiety disorders with an evidence base are psychological and behavioral. The impact of treating anxiety with cognitive behavioral therapy (CBT) on HRV has been reported in a number of studies, with varying results. Diveky, Prasko (115) reported that a 6-week CBT program on a sample of 31 panic patients had no significant impact on HRV. Mathewson, Schmidt (116) investigated the impact of 12 weeks of 2 h group CBT sessions in 23 patients with SAD, reporting a decrease in resting RSA levels over the course of the study. The authors note this reduction in RSA may have been due to the anticipation of a stressful task (i.e., anxiety provoking speech) during testing sessions. Two studies have investigated the impact of exposure based treatments on HRV in samples of flight phobics. Bornas et al. (117) investigated the impact of an exposure treatment on HRV in 20 patients, reporting that they exhibited a significant reduction (*p* < 0.01) in HF HRV after six sessions of computer-assisted or virtual reality exposure over 3 weeks, and curiously that patients with lower pre-treatment HRV responded better to treatment. In contrast to these findings, Busscher et al. (118) reported on the impact of exposure (two 1-h flights) on HRV in 50 flight phobics patients, observing a significant increase in RSA a finding associated with a large effect size, even after a relatively brief treatment. A possible explanation for this discrepancy could be the differential impact between virtual versus *in vivo* exposure on psychophysiological outcomes. Sack et al. (119) examined the effect of eye movement desensitization and reprocessing (EMDR) treatment on RSA in 11 patients with PTSD. Following an average of 4.7 (ranging from one to eight) EMDR sessions, there was no difference in pre-treatment and post-treatment RSA; however, there was a significant increase in RSA at 6 month follow-up. This reduction at follow-up was preceded by symptom reduction at post-treatment assessment.

Finally, concerning studies that have investigated the effect of concurrent psychotropic and non-psychotropic treatment on HRV, Prasko e al. (46) examined the effect of both CBT and SSRIs on HRV in 19 patients with PD. Following 18 group CBT sessions and pharmacotherapy, all HRV power spectra increased, with HF HRV increasing significantly. Garakani et al. (31) administered either 12 weeks of CBT alone or CBT with sertraline (an SSRI), and in treatment responders, CBT alone was associated with increases in HRV, but not the combined treatment. Clearly, there is considerable heterogeneity in treatment studies with respect to treatment type and the specific disorder being treated. Thus, it is unsurprising that there is considerable variation in reports on HRV outcomes in response to treatment, and it remains to be seen whether successful treatment of anxiety disorders will be paired with increases in HRV. However, given that both anxiety and low HRV predict adverse cardiovascular outcomes, we recommend that anxiety patients consider cardiovascular risk reduction strategies, such as exercise, smoking cessation, dietary change, and meditation, in conjunction with normal treatment.

This study has a number of advantages, including the application of a standardized meta-analytic methodology to assess the impact of anxiety disorders on HRV, examination of a range of HRV measures, the inclusion of moderator analyses examining the effect of frequently overlooked confounds including psychiatric and medical co-morbidity, and medication status, assessment of publication bias, and the application of strict inclusion/exclusion criteria for the selection of studies. However, some limitations of the present study are worth noting. There is a paucity of studies reporting on the effects of GAD, OCD, SAD, and SP, placing limits on the conclusions that can be drawn about the effect of these disorders on HRV. This is particularly true for conclusions about HRV and OCD. Only two studies reported on HRV and OCD, with one study report no significant reductions (47), and one study reporting significant reductions with the caveat that the result may have been related to the use of psychotropic medications (29). We also found evidence for study heterogeneity, although this issue was addressed in part, by conducting follow-up analyses to observe the effect of specific anxiety disorders on HRV. Further, patients with an anxiety disorder suffer from co-morbid depression in up to 60% of cases (120), and although the present results indicate that HRV of patients with psychiatric co-morbidity does not differ from those without such co-morbidity, it should be noted that depression and co-morbid anxiety has been associated with greater HRV reductions than depression alone (121), and depression and co-morbid anxiety increases risk of all-cause mortality and CVD two- to threefold (122). We observed a disproportionate number of studies on PD, highlighting the need for future studies on other anxiety disorders. There is also a need for future studies to consider the impact of evidence-based treatments for anxiety disorders on HRV, allowing for the impact of symptom reduction on HRV to be determined and downstream effects on health and well-being to be elucidated. Finally, there is a clear link both between anxiety-disease and HRV-disease, and these have been documented extensively by other authors. However, there are no prospective studies reporting on all three factors, and how HRV mediates the link between anxiety and disease. Future prospective studies examining the link between anxiety and disease while reporting on HRV will be valuable in this regard.

In summary, the present results have important implications for the long-term health and well-being of patients with a variety of anxiety disorders, considering the body of work highlighting a role for impaired vagal regulation as a risk factor for CVD and all-cause mortality [see Ref. (9, 10, 123) for reviews]. In conclusion, we advise clinicians to consider comprehensive cardiovascular risk reduction strategies for anxiety patients.

## Conflict of Interest Statement

The authors declare that the research was conducted in the absence of any commercial or financial relationships that could be construed as a potential conflict of interest.

## Supplementary Material

The Supplementary Material for this article can be found online at http://www.frontiersin.org/Journal/10.3389/fpsyt.2014.00080/abstract
