# Supplementary material for: Anxiety Disorders are Associated with Reduced Heart Rate Variability: A Meta-Analysis
Source: Front Psychiatry. 2014 Jul 11;5:80. doi: 10.3389/fpsyt.2014.00080 (PMC4092363; doi:10.3389/fpsyt.2014.00080)
Supplement: Supplementary file 1 [file Data_Sheet1.DOC]

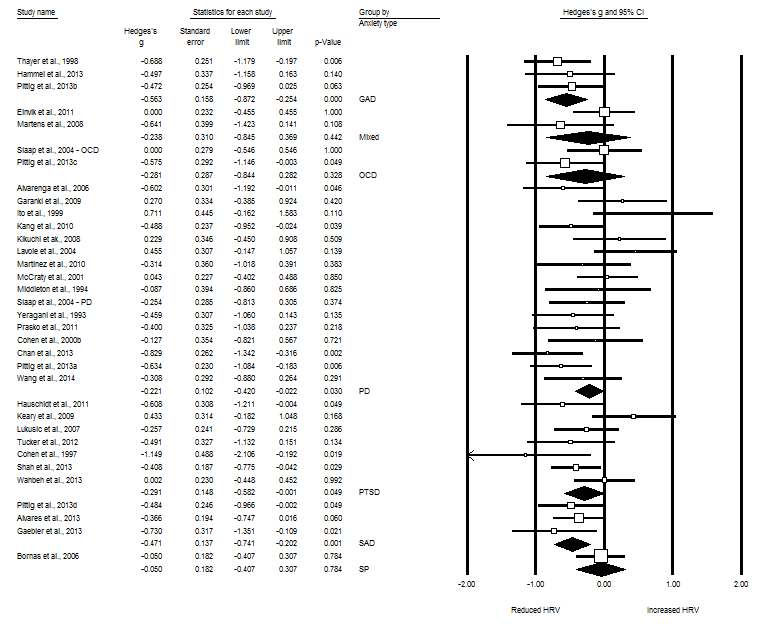


*S1. High Frequency HRV grouped by disorder*


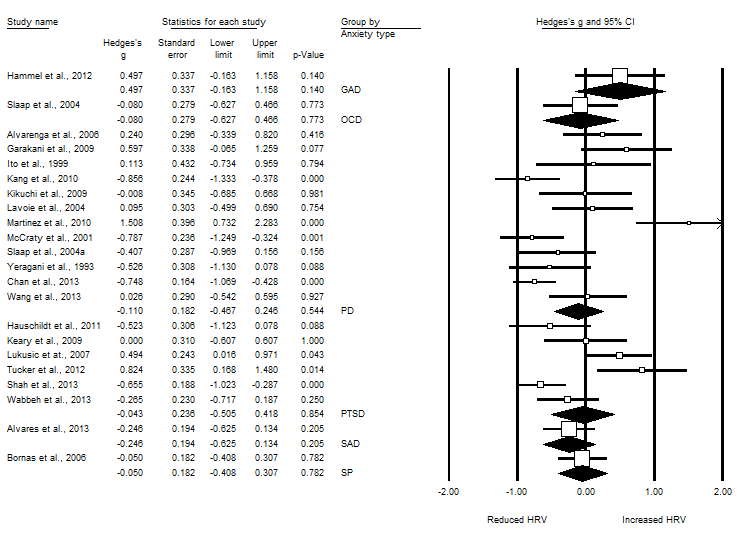


*S2. Low Frequency HRV grouped by disorder*

*
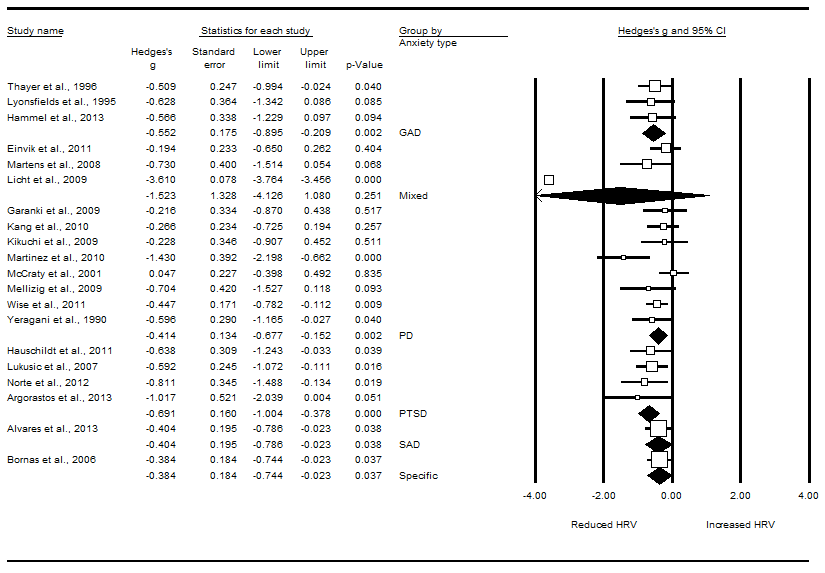
*

*S3. Time Domain HRV grouped by disorder (Licht et al., 2009, included)*

*
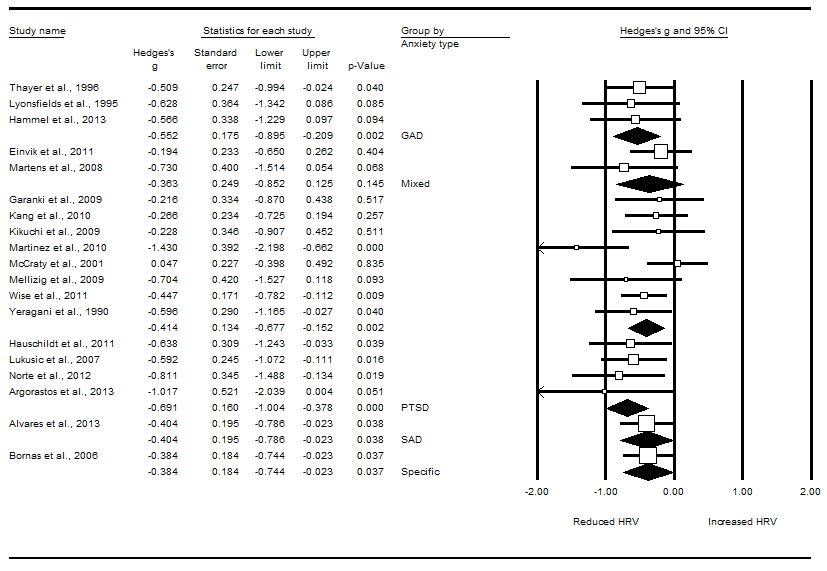
*

*S4. Time Domain HRV grouped by disorder (Licht et al., 2009, removed)*
